# PI3Kγδ inhibition suppresses key disease features in a rat model of asthma

**DOI:** 10.1186/s12931-024-02814-1

**Published:** 2024-04-23

**Authors:** James W. Pinkerton, Silvia Preite, Antonio Piras, Dimitrios Zervas, Thomais Markou, Mark S. Freeman, Tobias Hofving, Emil Ivarsson, Sara J. Bonvini, Wayne Brailsford, Linda Yrlid, Maria G. Belvisi, Mark A. Birrell

**Affiliations:** 1 Early Respiratory & Immunology, Biopharmaceuticals R&D AstraZeneca, Gothenburg, Sweden; 2 Respiratory Pharmacology group, Airway Disease section, NHLI, Imperial College, London, UK

**Keywords:** Phosphoinositide 3-kinase P110γδ asthma AZD8154

## Abstract

**Background:**

Two isoforms of Phosphoinositide 3-kinase (PI3K), p110γ and p110δ, are predominantly expressed in leukocytes and represent attractive therapeutic targets for the treatment of allergic asthma. The study aim was to assess the impact of administration of an inhaled PI3Kγδ inhibitor (AZD8154) in a rat model of asthma.

**Methods:**

Firstly, we checked that the tool compound, AZD8154, inhibited rat PI3K γ & δ kinases using rat cell-based assays. Subsequently, a time-course study was conducted in a rat model of asthma to assess PI3K activity in the lung and how it is temporally associated with other key transcription pathways and asthma like features of the model. Finally, the impact on lung dosed AZD8154 on target engagement, pathway specificity, airway inflammation and lung function changes was assessed.

**Results:**

Data showed that AZD8154 could inhibit rat PI3K γ & δ isoforms and, in a rat model of allergic asthma the PI3K pathway was activated in the lung. Intratracheal administration of AZD8154 caused a dose related suppression PI3K pathway activation (reduction in pAkt) and unlike after budesonide treatment, STAT and NF-κB pathways were not affected by AZD8154. The suppression of the PI3K pathway led to a marked inhibition of airway inflammation and reduction in changes in lung function.

**Conclusion:**

These data show that a dual PI3Kγδ inhibitor suppress key features of disease in a rat model of asthma to a similar degree as budesonide and indicate that dual PI3Kγδ inhibition may be an effective treatment for people suffering from allergic asthma.

**Supplementary Information:**

The online version contains supplementary material available at 10.1186/s12931-024-02814-1.

## Introduction

Asthma is a chronic, heterogenous, inflammatory disease of the airways which effects up to 300 million people worldwide [1]. Current therapies aim to achieve symptomatic control using ‘gold standard’ inhaled corticosteroids [2]. However, up to 50% of asthmatics do not achieve control despite high dose therapy, with high dose therapy associated with increased risk of unwanted side-effects [3–6]. As such, novel, inhaled therapies which are more targeted to the mechanisms driving the disease are warranted.

Phosphoinositide-3-kinase (PI3K) is a multifunctional lipid kinase, which plays a critical role in mediating a myriad of cellular functions such as proliferation, metabolism, and motility [7]. Although initially associated with cancer, aberrant activation of class I PI3Ks have been associated with several chronic respiratory diseases such as asthma [7–9]. Class I PI3Ks are activated by ligands binding to a G-protein coupled receptor (GPCR) or receptor tyrosine kinase (RTK), which leads to phosphorylation of the plasma lipid phosphatidylinositol-4-5-bisphosphate (PIP_2_) resulting in phosphatidylinositol-4-5-trisphosphate (PIP_3_) [7, 9, 10]. Proteins with pleckstrin homology domains like the protein kinases, Akt and phosphoinositide-dependent kinase (PDK)1 congregate at the sites of PI3K activation through direct binding to PIP_3_. Binding of Akt and PDK1 to PIP_3_ leads to phosphorylation of Akt by PDK1, inducing activation of several downstream mediators critical for growth and proliferation [7, 9].

Class I PI3Ks consist of catalytic subunit (p110) and a regulatory subunit. The class I p110 catalytic subunit has 4-distinct isoforms, with the p110γ and p110δ subunits being primarily expressed in leukocytes [7, 11, 12]. Both p110γ and p110δ play critical roles in the regulation of the innate immune and adaptive immune responses [11, 12]. The p110γ subunit is crucial for T-cell maturation and survival [13]. Studies have shown that the p110δ is crucial for mediating B-cell function, T-cell activation, and proliferation [14].

In the context of asthma, targeting PI3K is attractive as it plays a critical role in driving many of the pathophysiological features driving the disease. In vitro studies have shown that activation of PI3K plays critical roles in airway smooth muscle contraction, proliferation, and production of numerous chemokines [7, 15–17]. Furthermore, PI3K activation is also important in respect to mucus secretion from airways epithelial cells [18]. In respect to inflammation, PI3K activation is also crucial in driving granulocyte activation, degranulation, and cytokine release [19–21]. These observations have been further demonstrated functionally with in vivo models of asthma showing a crucial role for PI3K in driving airways hyperresponsiveness (AHR) & airways inflammation [22–24]. Interestingly, the PI3K pathway may be a useful target for severe asthma, as PI3Kγ deficient mice have reduced airway remodelling; and suppression of PI3K restored steroid sensitivity in a steroid-resistant model of asthma [22].

Various PI3K inhibitors are currently under investigation for use in asthma and other respiratory disorders [7, 25, 26]. Pan-PI3K inhibitors like wortmannin and LY294002 are inappropriate due to toxicity or poor pharmacokinetic (PK) profiles [25]. As such, focus has shifted to γ and/or δ isoform specific inhibitors [25]. Oral PI3Kγδ inhibitor, duvelisib has been investigated in clinical asthma trials which showed beneficial effects, but further development has not occurred potentially due to poor side effect profile [27]. Nemarisilib, is an inhaled PI3Kδ inhibitor which was recently evaluated in clinical trials with no improvement in asthma symptoms and associated increase in cough propensity [28]. These data may suggest that an inhaled dual inhibitor may be a better therapeutic approach.

Therefore, we aimed to assess the pharmacological potential of an inhaled PI3Kγδ inhibitor, AZD8154, in an established rat model of asthma by determining the impact on target engagement (TE), inflammation and the late asthmatic response (LAR).

## Methods

### In vitro assays of PI3Kγ & δ activation following administration of PI3Kγδ inhibitor, AZD8154

PI3K γ assay [29]: whole blood from male Sprague Dawley rat was collected in heparin coated tubes and pre-treated (1 hr., 37 °C, 5% CO_2_) with vehicle DMSO or PI3K inhibitors followed by stimulation with Rat CXCL1/CINC-1 (515-CN-050, R&D) at 500 ng/mL (EC_80_ determined in house; 1 hr., RT). Next, cells were stained with Fc block (550,273, BD bioscience, 1:100; 20 mins, RT), CD45R-B220-PeCy7 (HIS24) (25–0460-82, eBioscience, 1:100), CD43-PE (W3/13) (202,812, Biolegend, 1:100), HIS48-FITC (11–0570-82, eBioscience, 1:100), CD11b-APC (562,102, BD Bioscience, 1:100). Cells were lysed and fixed with BD FACS lysing solution (349,202, BD Biosciences; 45 mins, RT) before FACS acquisition (BD LSRFortessa™). Data were analysed using FlowJo 9.9 software (TreeStar). FACS gating strategy: lymphocytes (FSC-A vs SSC-A), doublets exclusion (FSC-W vs FSC-H), neutrophil identification B220-, HIS48hi, CD43hi, SSC-Ahi, geom. MFI for CD11b on neutrophils. IC_50_ values were determined in Graph Pad through a nonlinear fit on log transform data: log inhibitor vs response, variable slope, 4 parameters. Fraction unbound in diluted whole blood is calculated using the following equation: ${\boldsymbol{fu}}^{\prime }=\frac{\boldsymbol{DF}\cdot {\boldsymbol{fu}}_{\boldsymbol{b}}}{1+\left(\boldsymbol{DF}-1\right)\cdot {\boldsymbol{fu}}_{\boldsymbol{b}}}$ where fu’ is the fraction unbound in diluted whole blood; fu_b_ is the fraction unbound in undiluted whole blood; DF is the dilution factor. Fraction unbound in 100% whole blood (fu_b_) is calculated as fu_p_/BP, where fu_p_ is the experimentally determined fraction unbound in undiluted plasma and BP is the experimentally determined blood to plasma partition ratio of drug Table 1.

**Table 1:** Unbound fraction of compounds in whole blood

Compound	Rat ppb (fu, %)	Rat plasma fu	Rat B/P ratio	Rat fub	DF	fu’
**PI3Kγδ - AZD8154**	1.5	0.015	0.82	0.018	1.05	**0.0192**
**PI3Kδ - GSK2269557**	4.4	0.044	0.88	0.050	1.05	**0.0524**
**PI3Kγ - AZD3458**	15	0.15	0.75	0.200	1.05	**0.2079**

PI3K δ assay [30]: single cell suspension was obtained from the spleen of male Sprague Dawley rat; cells were cultured in RPMI containing heat-inactivated 10% FBS, pre-treated (1 hr., 37 °C, 5% CO_2_) with vehicle DMSO or PI3K inhibitors, followed by stimulation with AffiniPure F(ab’)_2_ Fragment Goat Anti-Rat IgM, μ chain specific, Jackson Immunoresearch (112–006-075) at 5 μg/mL (EC_80_ determined in house; 20–22 hrs). Then, cells were washed with FACS buffer (2% FBS, 2 μM EDTA) and stained (15 mins, 4 °C) with Fc block (1:100, 550,273, BD bioscience), LIVE/DEAD Fixable Aqua Dead Cell Stain Kit (L34957, Invitrogen, 1:300), CD45R (B220)-PeCy7 (HIS24) (25–0460-82, eBioscience, 1:100), CD3-AF647 (IF4) (201,408, Biolegend, 1:100), CD86-FITC (24F) (200,305, Biolegend, 1:50). Cells were acquired on the Flow Cytometer (BD LSRFortessa™) and analysed using FlowJo 9.9 software (TreeStar). FACS gating strategy: lymphocytes (FSC-A vs SSC-A), doublets exclusion (FSC-W vs FSC-H), live cells (Aqua-530-), B cell identification CD3- B220 + % of positive B cells for CD86. IC_50_ values were determined in Graph Pad through a nonlinear fit on log transform data: log inhibitor vs response, variable slope, 4 parameters. Fraction unbound in in vitro assay is calculated using the following equation: ${\boldsymbol{fu}}^{\prime }=\frac{\boldsymbol{DF}\cdot {\boldsymbol{fu}}_{\boldsymbol{p}}}{1+\left(\boldsymbol{DF}-1\right)\cdot {\boldsymbol{fu}}_{\boldsymbol{p}}}$ where fu’ is the fraction unbound in assay media; fu_p_ is the experimentally determined fraction unbound in undiluted human plasma; DF is the dilution factor (fold difference between protein concentration in human plasma and assay media, using an albumin concentration of 600 μM (40 g/L) for undiluted plasma) Table 2.

**Table 2:** Unbound fraction of compounds in whole blood

Compound	Human ppb (fu, %)	Human plasma fu	Fu’
**PI3Kγδ - AZD8154**	0.63	0.0063	**0.060**
**PI3Kδ - GSK2269557**	2.1	0.021	**0.177**
**PI3Kγ - AZD3458**	15	0.15	**0.638**

### Rat model of allergic asthma for time course characterisation of key disease features & treatment with corticosteroid, PI3Kγδ inhibitor, AZD8154

Male, adult, Brown Norway rats were obtained from Charles River Germany at a body weight range of 250-300 g. Animals were housed in temperature controlled individually ventilated cages in groups of 4 with food and water provided ad libitum. Animals underwent acclimatisation for at least 1 week prior to experimentation. Experiments were performed in accordance with the UK Home Office guidelines for animal welfare based on the Animals (Scientific Procedures) Act of 1986 and the ARRIVE guidelines (Fig. E2).

Initially a time course study was performed, followed by a characterisation study with compound & clinical comparator. For both studies, rats were sensitised on day 0, 14 and 21 by an intraperitoneal (i.p.) injection of 4 mL/kg (1 mL/rat) of ovalbumin (OVA;100 μg/rat) in Imject™ Alum (20 mg/rat aluminium hydroxide and 20 mg/rat magnesium hydroxide) or sham sensitised (Saline [Sal] in Imject™ Alum) [31, 32]. On day 28 rats were challenged with 1% (w/v) aerosolised OVA in isotonic saline, or sham challenged with isotonic saline (30 mins). For the compound study, on day 28, 1 hr. prior to challenge, some rats received intratracheal (i.t.) doses of AZD8154 (0.02, 0.1 or 0.3 mg/kg), budesonide (3 mg/kg) or vehicle under isoflurane anaesthesia (4% v/v) at 1 mL/kg (Fig. E2).

### Enhanced pause (Penh) in whole body plethysmography

In both studies, 30 mins post-challenge, rats were placed unrestrained in Perspex plethysmographs connected to bias flow pumps (DSI Europe Ltd), with food and water ad libitum. 55 mins post-challenge, a five mins baseline recording was taken and the average penh and area under the curve (AUC) was recorded at 10 mins intervals for a total duration of 5 hrs and was used for analysis (DSI Europe Ltd).

### Termination, blood sampling, preparation of plasma

For the time course study, animals were terminally anaesthetized, via overdose i.p. with sodium pentobarbitone (200 mg/kg), 1–24 hrs post antigen challenge. For the compound study, animals were terminally anaesthetised, via overdose i.p. with sodium pentobarbitone (200 mg/kg), 2 or 24 hrs post antigen challenge. For both studies, prior to completion of death, whole blood was attained via cardiac puncture with a heparinised syringe. Whole blood was centrifuged (1200×*g*, 10 mins, 4 °C) and plasma was collected and stored for future assessment (− 80 °C).

### Bronchoalveolar lavage (BAL) sampling and preparation of cytospin

Following blood collection, rats underwent tracheostomy and were cannulated. Rats were lavaged and bronchoalveolar lavage (BAL) fluid was collected (2 × 3 mL RPMI, 30 secs, 70% vol. retained). Slides were prepared using 100 μL of neat BAL, centrifuged (700 rpm, 5 mins, RT, low acceleration; Cytospin 2, Shandon, Runcorn, UK). 800 μL of neat BAL was collected and centrifuged (800×*g*, 10 mins, 4 °C). Supernatant was collected and stored (− 80 °C), and cell pellet was resuspended (200 μL, RPMI), total leukocytes were attained using a Sysmex XP-300 automated cell counter (Sysmex Ltd., Milton Keynes, UK). Remaining BAL was centrifuged (800×*g*, 10 mins, 4 °C) and supernatant collected and stored (− 80 °C).

### Lung collection

Following BAL collection, the post-caval lobes were isolated and removed, snap frozen and stored (− 80 °C).

### Cytospin staining & differential cell counts

BAL cytospin slides were placed in an automated stainer (Hematek 2000, Ames. Co., USA) and stained with a Modified-Wright stain (Siemens, Germany). Differential cell counts using a light microscope were performed based on morphological criteria were performed to determine percentages of macrophages/monocytes, lymphocytes, neutrophils & eosinophils. Total numbers of each distinct cell type were determined based on total leukocyte numbers.

### Target engagement & specificity

#### Whole protein extraction & Bradford assay

For assessment of protein levels and nuclear protein levels, the post-caval lobes were powderised in liquid nitrogen. For whole protein extraction, 30 mg of tissue was gently homogenised in Complete Lysis Bufer (Tris-Lysis Buffer, Protease Inhibitor Solution, Phosphatase Inhibitor I & II, 4 °C, 10 mins; MSD, USA). Samples were centrifuged (15,000×*g*, 20 mins, 4 °C).

5 μL of supernatant (1:50 dilution) was taken for Bradford Assay which was run according to manufacturers’ instructions (BioRad, USA). Briefly, a seven-point standard curve using BSA in PBS was prepared at 1 mg/mL-0.0156 mg/mL. Standards and sample were added to a 96-well plate and following addition of dye-reagent (1:5 dilution in H_2_O) samples were incubated (5 mins, RT). Absorbance was measured using a spectrophotometer and total protein values interpolated to standard curve (595 nm; Spectromax, UK).

#### pAkt (T308), p signal tranducer and activator of transcription (STAT)3 & pSTAT5a, b

10 μg of protein from lung tissue extract were utilized in pAkt (T308); pSTAT3, pSTAT4 & STAT5a, b (Phospho-STAT Panel kit) MSD Assay plates (Meso Scale Delivery, USA) and run according to manufacturers’ instructions. Arbitrary electrochemiluminescence signals were read, the blank subtracted and values determined in each plate using a MSD S-Plex plate reader (Meso Scale Delivery, USA).

#### Nuclear tissue extraction

Nuclear fractionations of whole lung tissue were extracted utilizing a Nuclear Extraction kit (Nuclear Extract Kit, ActiveMotif, USA). Briefly, 30 mg of powderised tissue was added to Wash Buffer A and gently mixed (120 μL, 10 mins, 4 °C). Samples were centrifuged (850×*g*, 10 mins, 4 °C) and supernatant was discarded. Cell pellet was then added to Buffer B and gently mixed (50 μL, 15 mins, 4 °C). Detergent was added (2.5 μL) and samples were vortexed (10 secs). Samples were then centrifuged (14,000×*g*, 1 min, 4 °C). The cytoplasmic fractionation was collected and frozen (− 80 °C). The nuclear pellet was resuspended in Buffer C (50 μL, 30 mins), with vortexing occurring every 10 mins. Samples were then centrifuged (14,000×*g*, 10 mins, 4 °C). Nuclear fractionation was collected and utilised for Bradford Assay to determine protein levels and for assessment of NF-κB p65 levels.

#### Nuclear factor (NF)-κB p65

10 μg (5 μL) of nuclear tissue extract were utilized in NF-κB p65 TransAm Activation Assays (Active Motif, USA) and run according to manufacturer’s instructions. Optical density was determined utilizing a spectrophotometer, the blank was subtracted, and values determined (450 nm; Spectromax, UK).

### Pharmacokinetic profile

#### Assessment of plasma levels of compounds

50 μL plasma was protein precipitated by addition of 180 μL acetonitrile containing 0.2% formic acid & 50 nM internal standard. After vortex mixing and centrifugation at 400×*g* for 20 mins, the supernatants were diluted 1:1 with 0.2% formic acid in water to match the initial mobile phase. Plasma concentrations of AZD8154 were determined by liquid chromatography with tandem mass spectrometric detection (LC-MS/MS).

#### Assessment of lung levels of compounds

Lung pieces were weighed and homogenised in a 1 mL ringer solution using bead-beating technology (Bertin Technologies, Montigny le Bretonneux, France). For the analysis, 50 μL of the lung homogenate was protein precipitated by addition of 180 μL acetonitrile containing 0.2% formic acid and 50 nM internal standard. After vortex mixing and centrifugation at 4000×*g* for 20 mins, the supernatants were diluted 1:1 with 0.2% formic acid in water to match the initial mobile phase. Lung concentrations of AZD8154 were determined by LC-MS/MS. The dilution step was accounted for when reporting the AZD8154 concentrations in lung.

#### Statistics

Comparisons between multiple groups were performed using a one-way ANOVA and an appropriate post-test or a nonparametric equivalent, as appropriate. Lung function data were assessed using a two-way ANOVA with an appropriate post-test. Analyses were performed using GraphPad Prism Software (San Diego, California, USA).

## Results

### PI3Kγδ inhibitor suppresses PI3Kγ and PI3Kδ pathway activation, in a rat cell-based assay

**Fig. 1:**
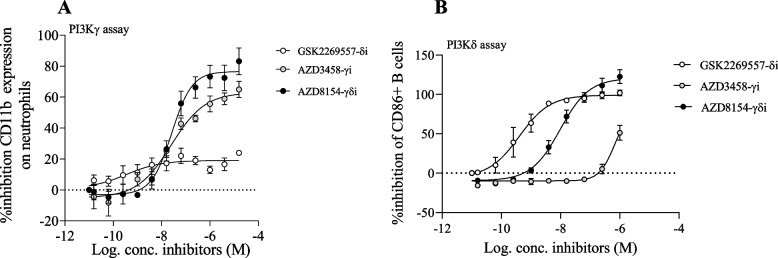
PI3Kγδ inhibitor, AZD8154, is able to inhibit both γ and δ isoforms of PI3K in rat cell-based assays. To determine if AZD8154 was able to inhibit rat PI3K isoforms rat blood neutrophils were pre-treated with AZD8154 and stimulated with CXCL1/CINC-1 to activate the PI3Kγ isoform (**A**); or rat splenic B cells were pre-treated with AZD8154 and stimulated with anti-IgM to activate the PI3Kδ isoform (**B**). Selective isoform inhibitors were included for reference and comparison (PI3Kγ inhibitor: AZD3458, and PI3Kδ inhibitor: GSK2269557). Data are presented as mean ± SEM. γ assay (**A**) & δ assay (**B**), pooled results from 3 independent experiments. Free IC_50_ (mean ± SEM) were calculated from *n* = 3 experiments by correcting for protein binding in the assays (**A**, **B**)

To determine if the PI3Kγδ inhibitor, AZD8154, was able to inhibit rat PI3Kγ and PI3Kδ isoforms, we utilised established rat in vitro assays. For comparison PI3Kγ or PI3Kδ inhibitors were included; AZD3458 and GSK2269557, respectively. The PI3Kγδ inhibitor, AZD8154, caused a concentration related inhibition in CD11b expression, a marker of PI3Kγ pathway activation (Fig. 1A; rat γ total IC_50_ = 28.7 ± 4.1 nM, free IC_50_: 0.54 ± 0.08 nM) and CD86 expression, a marker of PI3Kδ activation (Fig. 1B; rat δ total IC_50_ = 10.5 ± 6.2 nM, free IC_50_: 0.63 ± 0.37 nM). The reference inhibitors blocked their respective assay but, at the relevant concentration, had no impact on the other assay indicating the relative selectivity of the systems.

### Early activation of the PI3K pathway in lung was associated with increased airway inflammation and changes in lung function like the late asthmatic response (LAR) observed in some asthmatics

To determine whether the PI3K pathway is activated in the rat model of asthma, a time course study was performed to elucidate the activation in respect to phosphorylation of Akt (pAkt), a downstream marker of PI3K activation, in whole lung. We also assessed the activation status of two other key pro-inflammatory pathways: Janus kinase (JAK)/STAT and NF-κB and the resultant airway inflammation and the LAR.

**Fig. 2:**
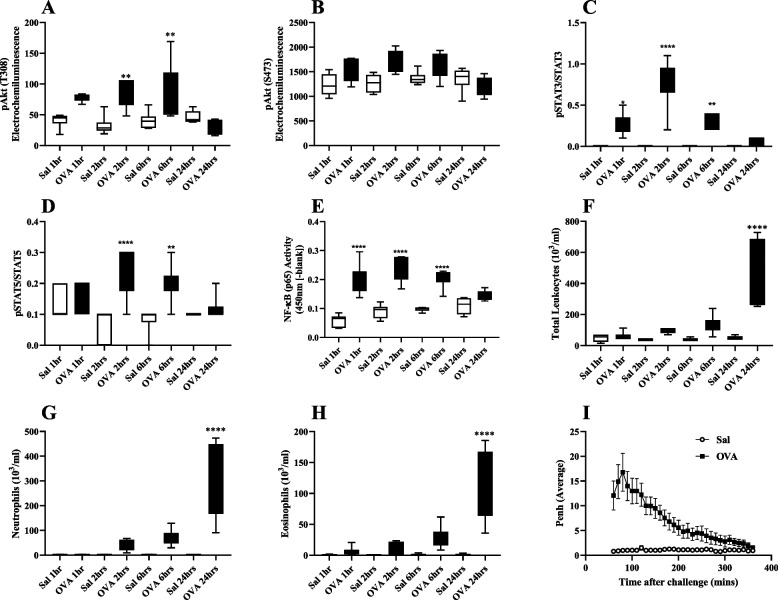
Activation of the PI3K, JAK/STAT and NF-κB pathways in lung is associated with increased cellular inflammation and the late asthmatic response (LAR). Akt phosphorylation, a downstream marker of PI3K pathway activation, was assessed after antigen challenge (OVA) in whole lung homogenate (**A** and **B**) pSTAT3 (**C**), pSTAT5a, b (**D**) and NF-κB (**E**) levels were assessed in whole lung homogenate and nuclear extract, respectively. Bronchoalveolar lavage (BAL) was taken to assess total leukocytes (**F**), neutrophils (**G**) & eosinophils (**H**) numbers. Whole body plethysmography in unrestrained rats was used to monitor the late asthmatic response (LAR; I). Data are presented as *boxes* (Q2 to Q3 with the median) and *whiskers* (min to max). Statistically significant differences between time matched, saline and OVA challenge groups is indicated with **p < 0.01 and ****P < 0.0001. Two experiments (*n* = 6 or 8)

OVA challenge resulted in a statistically significant increase in markers of the activation of PI3K, JAK/STAT and NF-κB pathways (Fig. 2A, C, D, E) compared to saline challenged, time matched groups.

OVA challenge results in a statistically significant increase in total leukocytes (Fig. 2F), neutrophils (Fig. 2G) and eosinophils (Fig. 2H) at 24 hrs post-challenge compared to saline challenged groups (OVA vs. Sal). Lastly, this antigen induced inflammation was associated with marked lung functional changes which is reminiscent of the LAR observed in allergic asthma (Fig. 2I).

### Effect of a PI3Kγδ inhibitor in the rat model of allergic asthma

**Fig. 3:**
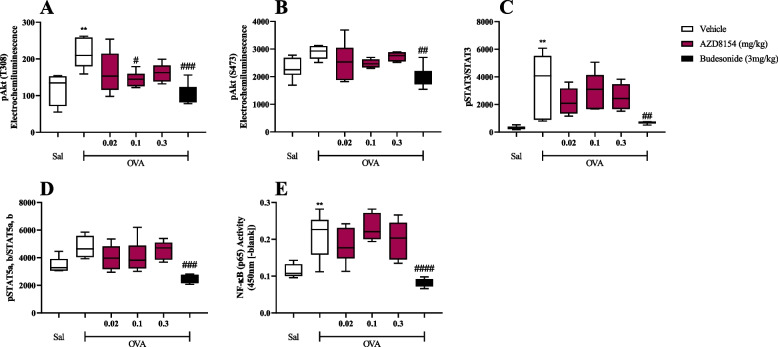
PI3Kγδ inhibitor, AZD8154, suppresses antigen induced increases in lung tissue pAkt levels, a downstream marker of PI3K activation. The impact of AZD8154 and budesonide on PI3K (**A** & **B**), JAK/STAT (**C** & **D**) and NF-κB (**E**) pathways was assessed in the lung tissue 2 hours after antigen (OVA) challenge. Data are presented as *boxes* (Q2 to Q3 with the median) and *whiskers* (min to max). Statistically significant differences between Saline/Vehicle and other groups as presented as **p < 0.01; statistically significant differences between OVA/Vehicle and other groups are presented as ##*p* < 0.01, ###*p* < 0.001 and ####*p* < 0.0001. One experiment (*n* = 5–6)

Administration of AZD8154 and budesonide into the lungs suppressed the antigen induced activation of the PI3K pathway. OVA challenge results in an increase in lung tissue pAkt compared to saline challenged groups (OVA/Veh vs. Sal/Veh, Fig. 3A & B).

Treatment with AZD8154 results in a dose related suppression in pAkt levels in the lung tissuse (Fig. 3A & B) Treatment with budesonide caused a dramatic inhibition of the PI3K pathway activation marker, pAkt (Fig. 3A & B).

Budesonide treatment also caused a marked suppression of both the JAK/STAT and NF-κB pathways whereas the PI3Kγδ inhibitor had minimal impact.

OVA challenge results in a statistically significant increase in pSTAT3 (Fig. 3C), pSTAT5a, b (Fig. 3D) & NF-κB p65 (Fig. 3E) compared to saline-challenged groups (OVA/Veh vs. Sal/Veh), with budesonide supressing pSTAT3 (Fig. 3C), pSTAT5a, b (Fig. 3D) & NF-κB p65 (Fig. 3E; OVA/Bud vs. OVA/Veh).

The suppression of the PI3K pathway by AZD8154 was associated with a reduction in airway inflammation and LAR.

**Fig. 4:**
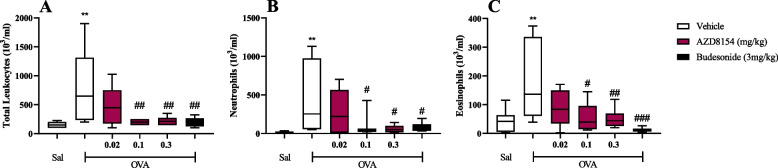
PI3Kγδ inhibitor, AZD8154, suppresses eosinophilia and neutrophilia in BAL. The impact of AZD8154 and budesonide on airways inflammation in BAL fluid collected 24 hours after antigen (OVA) challenge in respect to total leukocytes (**A**), neutrophils (**B**), eosinophils (**C**) was assessed. Data are presented as *boxes* (Q2 to Q3 with the median) and *whiskers* (min to max). Statistically significant differences between Saline/Vehicle and other groups as presented as **p < 0.01; statistically significant differences between OVA/Vehicle and other groups are presented as #*p* < 0.05, ##*p* < 0.01 & ###*p* < 0.001. One experiment (*n* = 7–8)

OVA challenge results in a statistically significant increase in total leukocytes (Fig. 4A), neutrophils (Fig. 4B) & eosinophils (Fig. 4C) compared to saline-challenged groups (OVA/Veh vs. Sal/Veh), with budesonide reversing these key features to basal levels (OVA/Bud vs. OVA/Veh).

Administration of AZD8154 results in a statistically significant decrease neutrophils & eosinophils (Fig. 4B, C) in a dose-dependent manner.

**Fig. 5:**
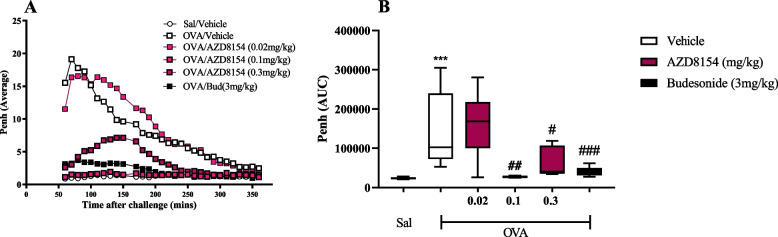
PI3Kγδ inhibitor, AZD8154, suppresses the LAR. Whole body plethysmography was used to assess LAR after antigen (OVA) challenge. Changes in penh (average) were monitored as a marker of antigen challenge induced LAR (1-6 hrs after treatment with vehicle, AZD8154 or budesonide (**A**). The data is represented as AUC (**B**). Data are presented as mean (A) & *boxes* (Q2 to Q3 with the median) and *whiskers* (min to max; B). Statistically significant differences between Saline/Vehicle and other groups as presented as ***p < 0.001; statistically significant differences between OVA/Vehicle and other groups are presented as #*p* < 0.05, ##*p* < 0.01 & ###*p* < 0.001. One experiment (*n* = 7–8)

OVA challenge results in a statistically significant increase in LAR (Penh; Fig. 5A, B) compared to saline challenged groups (OVA/Veh vs. Sal/Veh) with budesonide inhibiting the lung function change (OVA/Bud vs. OVA/Veh).

Administration of AZD8154 results in a statistically significant dose-dependent decrease in LAR (Fig. 5A, B).

The concentration of AZD8154 in lung tissue and plasma samples were determined in all groups dosed with AZD8154. Mean AZD8154 concentration in lung tissue and plasma increased with increasing dose at both time points; 2 & 24 hrs post-challenge (Fig. E1). The level of AZD8154 was significantly higher in the lung tissue and displayed greater retention in the lung tissue up to 24 hrs post-dosing compared to plasma levels (Fig. E1).

## Discussion

Class I PI3Ks have emerged as attractive therapeutic targets in chronic inflammatory disorders. Initially conceived of as a therapeutic target in cancer, several studies demonstrated a key role for PI3K pathway activation in driving features of disease in asthma [7, 9, 25]. Experimental studies have demonstrated that PI3K inhibition with pan-blockers like LY294002 and wortmannin suppress features of disease, however, pan-PI3K inhibitors like these are unattractive clinically due to their toxicity profiles [25]. PI3Kα & β isoforms also play critical roles in normal cellular responses, with inhibition of these isoforms resulting in embryonic lethality in mice [33, 34]. Therefore, current strategies in chronic inflammatory disorders look at the development on PI3Kγ and/or δ specific inhibitors.

Current approved PI3Kδ and PI3Kγδ inhibitors, idelasib and duvelasib, respectively, are orally delivered compounds for the treatment of chronic lymphocytic leukopenia, however, these are inappropriate in asthma due to their toxicity profiles [27, 35]. As such, current focus has looked at the development of inhaled therapies where systemic side-effects are less likely [26, 28]. Nemiralisib is an inhaled PI3Kδ specific inhibitor, investigated in asthmatics, COPD patients and PI3Kδ syndrome, however, development has been suspended due to lack of efficacy and increased incidence in cough [28]. Our study investigated the potential of an inhaled PI3Kγδ inhibitor, AZD8154, in a rat model of asthma to determine if it could modulate key disease features.

To ensure the activity of AZD8154 crossed to the rat kinases and thus could be used in the rat model, we tested AZD8154 against established γ, AZD3458, & δ, GSK2269557 specific inhibitors in rat cell-based systems.

Having demonstrated that AZD8154 could inhibit rat PI3K isoforms, we tested the compound in the rat model. This model of allergic asthma mimics some features of human allergic asthma such as increased eosinophilic and neutrophilic cellular inflammation in the lung and LAR [31, 32, 36]. Further to this, this model demonstrates a LAR as observed by visual assessment and non-invasive lung function, which is attenuated by anaesthesia [31, 32, 36].

Drug metabolism & pharmacokinetic (DMPK) analysis after administration of AZD8154 into the lungs showed good retention withing the airways and low levels systemically, suggesting that any modulation of the rat model phenotype was primarily due to local lung blockade of the PI3K axis. Indeed, our data showed that AZD8154 did suppress the antigen challenge induced activation of this pathway in the lung tissue. Interestingly, whilst the pan anti-inflammatory corticosteroid, budesonide, inhibited not only the PI3K axis but also the JAK/STAT and NF-κB pathways, the impact of AZD8154 appear to be predominantly on the PI3K pathway. These data demonstrate that our compound is target specific and that in an in vivo setting the inhibition of PI3K does not impact on the JAK/STAT and NF-κB pathways, unlike budesonide. And yet both AZD8154 and budesonide inhibited the cellular inflammation in the airways and the LAR associated with antigen challenge in this model. This would suggest that just targeting PI3Kγδ has the same beneficial effect as a steroid but without the global suppression of immune mechanisms thought to be associated with some of the unwanted effects of chronic steroid treatment [3–6].

## Conclusions

In summary, our data demonstrate that administration of AZD8154 into the airways can suppress inflammation and LAR in a rat model of allergic asthma. Overall, our data suggests that the inhibition of PI3Kγδ in the lungs could be beneficial to patient suffering with allergic asthma.

### Supplementary Information

**Supplementary Material 1.**

## Data Availability

Data is provided within the manuscript or supplementary information files

## Abbreviations

AAD: Allergic airways disease
AHR: Airways hyperresponsiveness
Alum: Aluminium hydroxide
BAL: Bronchoalveolar lavage
BN: Brown Norway
Bud: Budesonide
DMPK: Drug metabolism & pharmacokinetics
FBS: Fetal Bovine Serum
GPCR: G-protein coupled receptor
IC_50_: Half maximal inhibitory concentration
IL: Interleukin
IT: Intratracheal
JAK: Janus kinase
LAR: Late Asthmatic Response
LLOQ: Lower limit of quantification
NF-κB: Nuclear factor κB
OVA: Ovalbumin
Penh: Enhanced pause
PDK: Phosphoinositide-dependent kinase
PI3K: Phosphoinositide 3-kinase
PIP_2_: Phosphatidylinositol-4-5-bisphosphate
PIP_3_: Phosphatidylinositol-4-5-trisphosphate
RPMI: Roswell Park Memorial Institute
RTK: Receptor tyrosine kinase
Sal: Saline
STAT: Signal transducer and activator of transcription
TE: Target engagement
Veh: Vehicle
WBP: Whole body plethysmography
