# Supplementary material for: PI3Kγδ inhibition suppresses key disease features in a rat model of asthma
Source: Respir Res. 2024 Apr 23;25:175. doi: 10.1186/s12931-024-02814-1 (PMC11040934; doi:10.1186/s12931-024-02814-1)
Supplement: Supplementary file 1 [file 12931_2024_2814_MOESM1_ESM.odt]

Title: PI3Kγδ inhibition suppresses key disease features in a rat model of asthma

Authors:  James W. Pinkerton1, 2, Silvia Preite1, Antonio Piras1, Dimitrios Zervas1, 2, Thomais Markou1, 2, Mark S. Freeman1, 2, Tobias Hofving1, Emil Ivarsson1, Sara J. Bonvini1, 2, Wayne Brailsford1, Linda Yrlid1, Maria G. Belvisi1, 2, Mark A. Birrell1, 2

1Early Respiratory & Immunology, Biopharmaceuticals R&D, AstraZeneca, Gothenburg, Sweden

2Respiratory Pharmacology group, Airway Disease section, NHLI, Imperial College London, United Kingdom

Corresponding author: Mark A. Birrell, Executive Director, Early Respiratory & Immunology, Biopharmaceuticals R&D, AstraZeneca, Gothenburg, SE-431 83, Sweden Email: mark.birrell@astrazeneca.com

Supplementary Figures:

Figure E1. PI3Kγδ inhibitor, AZD8154, shows greater retention in the lung than plasma. Lung tissue (A) & plasma (B) PK concentrations of AZD8154 were determined 2hrs & 24hrs post-dosing. PI3Kγ & PI3Kδ IC50 are based threshold levels included are based on rat in vitro assay data (Fig. 1) Results are represented as ±SD. One experiment (n=5-8).  

Figure E2. Rat model of experimental asthma with budesonide (Bud), PI3Kγδ inhibitor, AZD8154, treatment. On day 0, 14 and 21, rats were sensitised (intraperitoneally, i.p.) with ovalbumin (OVA) and Alum™ (aluminium hydroxide/magnesium hydroxide) and challenged with aerosolised OVA (1% w.v.) or saline on day 28 to induce experimental asthma. Some groups of rats were also treated (intratracheally, i.t.) with Bud or AZD8154 1hr prior to challenge on day 28. Endpoints were assessed 1-24hrs following final challenge.
